# Colonies for Epileptic Patients

**Published:** 1897-03

**Authors:** 


### COLONIES FOR EPILEPTIC PATIENTS.

AT A MEETING of the Ontario Medical Association, June, 1894, Dr. A. McKinnon, of Guelph, introduced a resolution calling attention to the necessity for an institution in Ontario solely for the care of epileptics. Attention had previously been called to the subject in the annual reports of several of the medical superintendents of this province, and a deputation was appointed to place the matter before the Ontario government. Since then, in the state of New York, the Craig Colony for Epileptic Patients has been established, and its success has been such as to reflect great credit upon its projectors and to give additional hope for this very afflicted class. The colony now cares for 200 patients, or about one-fourth of those in the state needing care. Remarkable improvement is noticeable in those already admitted. Nearly every patient has gained in weight and in general health, and in all cases seizures have diminished in frequency. A school has been established, and various industries, as carpentry, sewing, painting, blacksmithing, are carried on. Patients of both sexes work in the field and garden, and 83 per cent. of males and 76 of females have been employed eight hours each day. One-half of the cost of maintenance has been produced by the colony. A similar colony has been established in Massachusetts, and its success is equally satisfactory. Ohio has made separate provision also for this class. Of the 30,000 or 40,000 epileptics in England, 1,100 are now cared for at Passmore Edwards House, a colony established in 1895. In the Province of Ontario there are, according to our asylum reports, over 300 epileptics in the several provincial institutions, besides the much larger number of similar cases which are taken care of at home.—*Canadian Practitioner*, February, 1897.

